# Early Biogeography of Otophysi Points to the Neotropics as the Cradle of Characiphysan Fishes

**DOI:** 10.1002/ece3.72431

**Published:** 2025-11-15

**Authors:** Achille Lenglin, Max Hidalgo, Guido Miranda, Aaron De la Sota, Pierre Caminade, Khalid Belkhir, Olga Otero, Pierre‐Olivier Antoine, Carmen Garcia‐Davila, Nicolas Hubert

**Affiliations:** ^1^ ISEM, Univ Montpellier, CNRS, IRD Montpellier France; ^2^ Departamento de Ictiología Museo de Historia Natural, Universidad Nacional Mayor de san Marcos Lima Peru; ^3^ Wildlife Conservation Society, Bolivia Program La Paz Bolivia; ^4^ Laboratorio de Limnología, Unidad de Ecología Acuática, Instituto de Ecología, Universidad Mayor de San Andrés, Campus Universitario La Paz Bolivia; ^5^ Observatorio de la Biodiversidad de la Amazonia Peruana (OBAP) Iquitos Peru; ^6^ Laboratoire Paléontologie Evolution Paléoécosystèmes Paléoprimatologie (PALEVOPRIM), UMR‐CNRS 7262, Université de Poitiers Poitiers France; ^7^ Laboratorio de Biología y Genética Molecular (LBGM) Instituto de Investigaciones de la Amazonía Peruana (IIAP) Iquitos Peru

**Keywords:** ancestral area reconstruction, biogeography, characiformes, freshwater fishes, gymnotiformes, phylogeny, Siluriformes

## Abstract

Freshwaters represent less than 1% of Earth's surface and only 0.02% of the available aquatic habitable volume, yet they host nearly half of the 35,500 known species of bony fishes. Ostariophysan fishes account for 70% of all freshwater fish diversity, comprising approximately 12,000 species across five highly speciose orders. They represent a major evolutionary radiation, the internal phylogenetic relationships of which remain the subject of intense debate. To better understand their early evolutionary history and origin, we reconstructed their phylogeny using dense taxonomic sampling and a combined dataset of complete mitochondrial genomes and sequences from four nuclear genes. Phylogenetic relationships and divergence times were inferred using Bayesian and Maximum Likelihood approaches and molecular dating analyses on a dataset of 687 ostariophysan species, comprising 21,701 aligned positions, including 15,707 variable sites. We also applied model‐based Maximum Likelihood ancestral area reconstruction to investigate the early evolutionary history of Otophysi. Our analyses yielded a highly supported phylogenetic hypothesis for Otophysi, highlighting the role of plate tectonics in driving multiple divergence events, along with subsequent range shifts. These findings are further supported by the contraction of the tropical belt, which began at the end of the Cretaceous and continued throughout the Paleogene. Our results support the divergence of Cypriniformes and Characiphysi as a consequence of the breakup of Laurasia and Gondwana. The origin of Characiphysi is traced to West Gondwana, and the subsequent expansion of the group cannot be explained without invoking transcontinental dispersal during the Upper Cretaceous‐Paleocene.

## Graphical abstract

Otophysan fishes represent 70% of living freshwater fishes with 12,000 species. Relationships among their four orders were explored through a dense taxonomic sampling including 529 complete mitochondrial genomes and 4 nuclear genes for 324 species. The resulting supermatrix including 21,701 base pair for 687 Ostariophysan fishes yielded highly supported relationships and ancestral area reconstructions point to the Neotropics as the craddle of Characiphysan fishes.

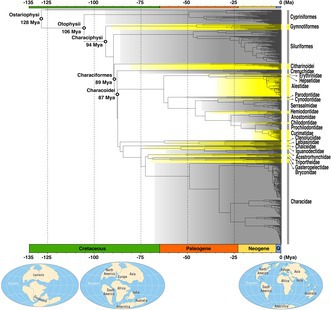

## 1 Introduction

With ca. 35,500 species belonging to 538 families and 56 orders, and 200 newly described species per year on average (Eschmeyer et al. 2025), bony fishes represent the most speciose group of vertebrates (The Catalog of Life 2025). Thriving in all aquatic habitats, from 3000 m deep in oceans to 4000 m high in mountains, fishes have evolved an astonishing diversity of form and life history strategies (Helfman et al. 2009; Nelson et al. 2016). This ample diversity, however, is not evenly distributed as freshwaters represent less than 1% of Earth's surface and only 0.02% of available aquatic habitable volume while hosting nearly half of known species of bony fishes (Dawson 2012; Tedesco, Beauchard, et al. 2017; Tedesco, Paradis, et al. 2017). Referred to as the freshwater fish paradox, this apparent contradiction has caught the attention of evolutionary biologists for decades (Levêque et al. 2008). The higher fragmentation and the lesser instability of freshwater habitats compared to oceans have been frequently invoked to account for this asymmetrical distribution of fish diversity on land and oceans (Tedesco, Paradis, et al. 2017). The increasing use of dated phylogenetic trees to explore fish diversity patterns, however, provided little support in favor of higher speciation and diversification rates in rivers compared to oceans (Miller 2021), despite a strong signature of a latitudinal effect in oceans (Miller et al. 2018; Rabosky et al. 2018). Instead, time spent in riverine and marine habitats over the 200‐million‐year history of bony fishes likely explains the freshwater paradox, freshwaters having concentrated a diversity inherited from a long evolutionary history across wider spatial scales in the past (Miller 2021; Miller and Román‐Palacios 2021).

**FIGURE 1:**
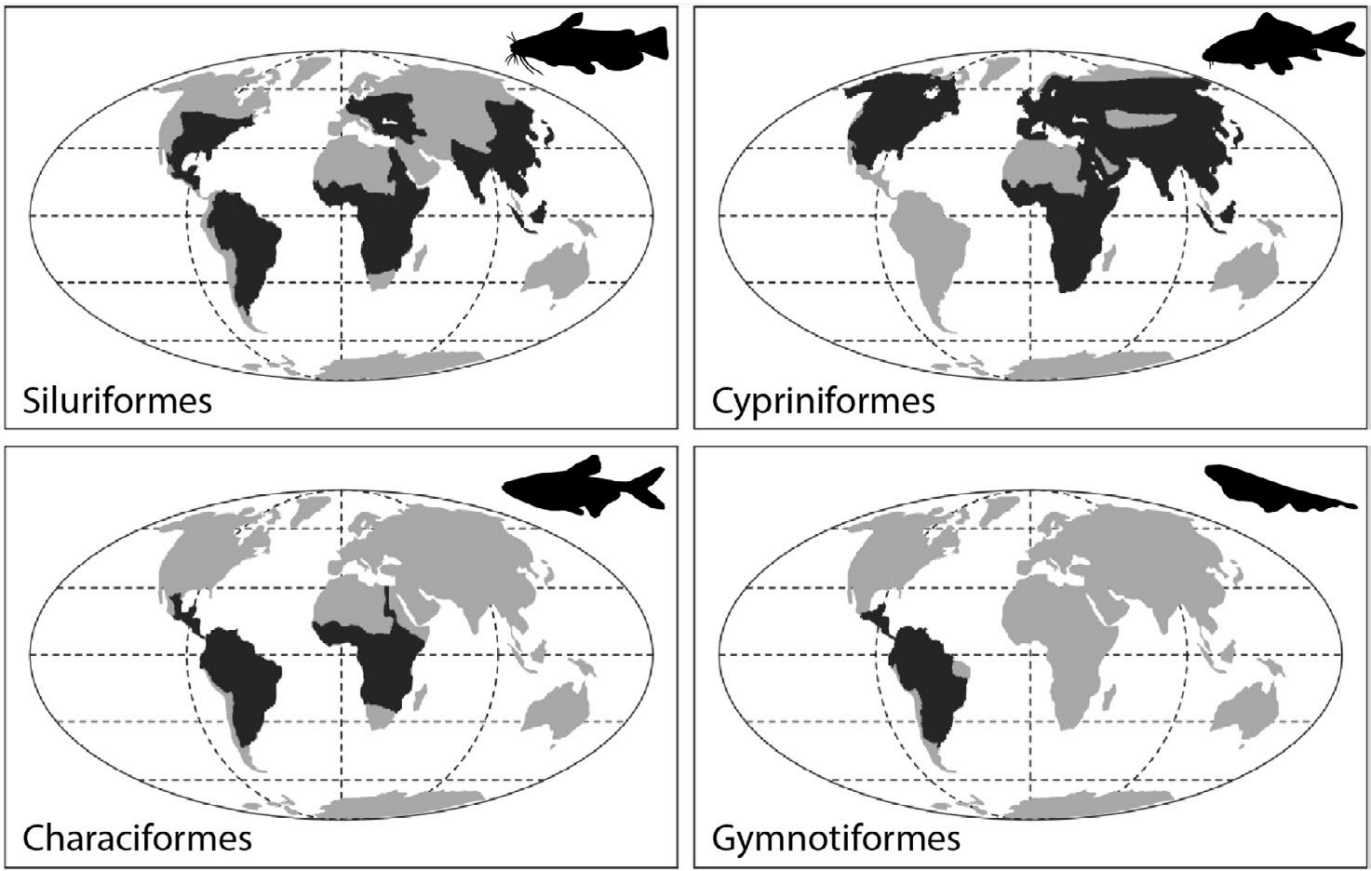
Distributions of the four orders of Ostariophysan fishes, modified from Chen et al. (2013).

In freshwaters, the superorder Ostariophysi (or ostariophysans) represents 70% of all freshwater fishes with ca. 12,000 species distributed in 1365 genera and 101 families, and is present in all continents except Antarctica (Nelson et al. 2016; Betancur et al. 2017; Eschmeyer et al. 2025). Further subdivided into two clades including Anatophysi (Gonorhynchiformes) and Otophysi (all remaining orders), the latter represents 99.5% of the diversity. Characterized by the presence of the Weberian apparatus, an osteological structure connecting the swim bladder to the auditory system and resulting from the merging of the first anterior vertebrae (Fink and Fink 1981; Nelson et al. 2016), otophysan fishes represent one of the most successful radiations of vertebrates. Including emblematic orders of freshwater fishes such as Cypriniformes (carps, minnows, and suckers), Siluriformes (catfishes), Characiformes (piranhas, pacus, tetras, and pencil fishes), and Gymnotiformes (knifefishes), they present a vast array of life history strategies (Winemiller 1989; Matthews 2012). The geographic distribution of each order, however, is markedly distinct, as Cypriniformes are widely distributed in North America and Eurasia, Characiformes are restricted to South America and Africa, Gymnotiformes are endemic to South America, and Siluriformes are distributed worldwide (Figure 1) (Nelson et al. 2016; Froese and Pauly 2023). The evolutionary history and biogeography of otophysan fishes have puzzled biologists for decades. Because of their geographic distribution (Nelson et al. 2016), fragmentary fossil record (Gayet 1982; Fink et al. 1984; Arratia and Cione 1996; Lundberg 1998; Lundberg et al. 1998; Antoine et al. 2016; Mayrinck et al. 2017) and difficulties in reconstructing their phylogenetic relationships (Alves‐Gomes et al. 1995; Orti 1997; Saitoh et al. 2006; Sullivan et al. 2006; Mayden et al. 2009; Betancur et al. 2019), their origin and early evolutionary history are still under debate (Lundberg 1993; Betancur et al. 2019; Melo et al. 2022).

The position of Anatophysi (i.e., Gonorhynchiformes) as the sister lineage of Otophysi (i.e., Cypriniformes, Gymnotiformes, Siluriformes and Characiformes), and the phylogenetic placement of Cypriniformes, sister to the remaining orders, are well supported (Arcila et al. 2017; Betancur et al. 2017; Chakrabarty et al. 2017; Dai et al. 2018; Hughes et al. 2018; Rabosky et al. 2018). The conundrum lies in the deep relationships among Characiphysi (i.e., Characiformes, Siluriformes and Gymnotiformes), and particularly in the position of the African superfamily Citharinoidei (i.e., Distichodontidae and Citharinidae), currently embedded in the order Characiformes but whose phylogenetic placement is uncertain in recent molecular studies (Betancur et al. 2018; Melo et al. 2022). The monophyly of Characiformes has been recently questioned by several molecular studies (Chakrabarty et al. 2017; Dai et al. 2018; Melo et al. 2022) which contrasts with others supporting the placement of the Citharinoidei sister to Characoidei (Arcila et al. 2017; Betancur et al. 2018; Hughes et al. 2018; Rabosky et al. 2018). Surprisingly, the implementation of phylogenomic strategies based on hundreds of nuclear markers to explore these relationships led to strong support for conflicting hypotheses, indicating that incongruence remains a problem in the era of big genomic data (Betancur et al. 2018). Pitfalls of phylogenomic approaches include poor filtering of paralogous genes, contamination during the construction of the genomic libraries and poor taxonomic sampling (Philippe et al. 2011, 2017; Shen et al. 2017; Betancur et al. 2018; Zhang et al. 2020). This is particularly true for groups known to be recalcitrant because they are ancient, very diverse and have experienced explosive radiations, such as Characiphysi, which started diversifying during Early Cretaceous times (Melo et al. 2022), encompasses ca. 12,000 species (Eschmeyer et al. 2025), and presents short internal branches (Betancur et al. 2018).

We explored the phylogenetic signal in mitochondrial and nuclear sequence data with the objective to examine phylogenetic patterns in the early branching of the Characiphysi tree and reconstruct ancestral area to explore the early biogeography of otophysan fishes. In this context, we opted to use complete mitochondrial genomes combined with multiple nuclear markers generated by Sanger sequencing (Rabosky et al. 2018), an approach which has remained largely unexplored for reconstructing the early phylogenetic relationships within Ostariophysi, despite each genomic compartment having been used independently in previous studies (Nakatani et al. 2011; Chen et al. 2013). These molecular markers present several advantages which, if combined, may help in advancing phylogenetic reconstructions in Characiphysi: (1) mitochondrial genes display little paralogs, and cases of nuclear integration of mitochondrial genes concern fragments not exceeding 500 base pairs (Zhang and Hewitt 1996; Bensasson et al. 2001), (2) mitochondrial genomes have a high resolving power in phylogenetic inferences of deep phylogenetic relationships in bony fishes due to their fast evolutionary rates and high number of variable positions (Miya et al. 2001; Saitoh et al. 2003; Yamanoue et al. 2007; Miya and Nishida 2015), (3) multiple mitochondrial genomes have been already produced for a vast array of ostariophysan lineages (see this study) which are still awaiting to be used in this context, (4) numerous sequences of nuclear markers produced by Sanger sequencing are now readily available to examine deep phylogenetic relationships among bony fishes and already proved to carry phylogenetic signal in deep time (Rabosky et al. 2018), (5) nuclear and mitochondrial genomes have markedly distinct evolutionary histories, and combining them is expected to yield robust inferences as phylogenetic signal is additive.

In the present study, we focused on the relationships among otophysan orders, with particular emphasis on the monophyly of Characiformes and the phylogenetic placement of Citharinoidei within Characiphysii. This focus stems from the fact that most recent controversies regarding the deep phylogenetic relationships among characiphysan orders have arisen from the unstable placement of Citharinoidei across studies. In this context, we mined all available mitochondrial genomes in Genbank for ostariophysan fishes, and complemented the taxonomic coverage by producing new mitogenomes for poorly covered Characiformes lineages. In parallel, available nuclear markers in Ostariophysi compiled in Rabosky et al. (2018) were selected. These data sets were separately and jointly analyzed to produce a dated time tree based on fossil calibrations further used to reconstruct ancestral areas among otophysan fishes. We focused on the early fragmentation of Pangea, specifically the separation of Laurasia in the north and Gondwana in the south, and its consequences for the evolutionary history of otophysan fishes. We also examined how subsequent continental separations influenced the diversification of characiphysan fishes.

## 2 Materials and Methods

### 2.1 Genomic Resources and Taxonomic Sampling

Two data sets were assembled here and subsequently combined including a set of complete mitochondrial genomes and four nuclear genes including myh6, rag1, rag2 and sh3px (Table S1). The nuclear genes come from the study by Rabosky et al. (2018), and were already filtered for rogue sequences corresponding to misidentification and/or paralogs. Only four nuclear genes were selected out of 21, which correspond to those with taxonomic coverage above 20% for ostariophysan fishes (Rabosky et al. 2018). The mitogenomes analyzed here come from different sources. First, previously published mitogenomes that were already assembled and annotated were downloaded from NCBI GenBank. Second, we inspected libraries of raw Illumina sequencing reads from previous studies using Ultra Conserved Element (UCE) to extract mitogenomes whenever enough reads were present to assemble a scaffold of at least 12,000 base pairs. For this, SRA files were downloaded from NCBI GenBank or ENA (Table S1). Third, new mitogenomes were produced for Characiform lineages which are poorly covered in available mitochondrial genomes in international repositories to complement mitogenomes coming from the two previous sources (Table S1).

For newly generated mitogenomes, total genomic DNA was extracted using the Macherey‐Nagel Nucleospin extraction kit following the manufacturer's specifications. The preparation of genomic libraries for mitogenome skimming (Straub et al. 2012; Dodsworth 2015) and sequencing on an Illumina Novaseq lane was conducted at Novogene (Cambridge, UK) with 150 bp paired‐end sequencing. Mitogenomes were assembled using the De Novo procedure implemented in Megahit (Li et al. 2015) and mitochondrial scaffolds were selected using MitoZ (Meng et al. 2019). Assembled mitogenomes were finally annotated using the online tool MitoAnnotator (Iwasaki et al. 2013) available at mitofish.aori.u‐tokyo.ac.jp. Annotated mitogenomes are accessible in NCBI GenBank (Table S1). Once protein‐coding genes were delimited, sequences were translated into amino acids to check for stop codons or insertion/deletion in codons with Aliview (Larsson 2014).

Combining mitochondrial and nuclear data sets, we were able to assemble sequences for 687 Ostariophysan species, including 529 Characiformes (23 of 24 families are covered), with two Gonorynchiformes (half of families), 41 Cypriniformes (67% of the families are covered), 17 Gymnotiformes (100% of families are covered) and 98 Siluriformes (70% of families are covered). This dataset represents the most extensive taxonomic sampling to date aimed at resolving otophysan relationships. As such, it is expected to improve phylogenetic reconstructions by mitigating the effects of long‐branch attraction through dense taxon sampling (Bergsten 2005; Philippe et al. 2005; Wiens 2005).

### 2.2 Phylogenetic Reconstructions

Mitogenomes were aligned with the multiple alignment program MUSCLE (Edgar 2004). Genes partitions were defined using annotations available in NCBI GenBank for six species scattered across the Characiformes tree of life including: *Psalidodon paranae* (GenBank KX609386.1), *Carnegiella strigata* (AP011983.1), *Megaleporinus piavussu* (KM886569.1), *Myleus* sp. (AP011997.1), *Oligosarcus argenteus* (MF805814.1) and *Phenacogrammus interruptus* (AB054129.1) (Table S2). Regions with a higher substitution rate (i.e., control region), affected by molecular saturation and presenting poorly resolving power over deep times (Miya and Nishida 2000), and those with single and large insertion (i.e., above 50 bp) were discarded from subsequent phylogenetic analyses. Besides, the rearrangement of genes observed in a few Characidae species between the ND2 and COI genes was also discarded (Xu et al. 2021). For nuclear genes, readily aligned sequences from Rabosky et al. (2018) were used for subsequent analyses. Datasets were ultimately combined, with missing genomes coded as missing data (‘?’) in cases where mitochondrial and nuclear data did not overlap.

The phylogenetic analyses were carried out using a partitioned ML method as implemented in IQtree v1.6.12 (Nguyen et al. 2015). A total of 16 partitions were defined for mitochondrial genomes, including 13 partitions for protein‐coding genes, two for ribosomal genes and one for all tRNAs, and four partitions were used for nuclear genes (Table S1). The most likely models for each partition were jointly identified using ModelFinder (Kalyaanamoorthy et al. 2017), as implemented in IQtree. The best models were selected according to the Bayesian information criterion (BIC) (Neath and Cavanaugh 2012). These analyses were conducted with all partitions sharing the same set of branch lengths and free rate heterogeneity. Missing nucleotide positions were coded in “N” and missing sequences (i.e., mitogenomes or nuclear genes) were treated as missing data and the corresponding positions were coded as “?”. Statistical support of the phylogenetic reconstructions was estimated with 5000 replicates of ultrafast bootstrap. All calculations were performed at the MBB platform (https://www.labex‐cemeb.org/en/montpellier‐bioinformatics‐and‐biodiversity‐mbb).

### 2.3 Fossil Record Used for Time Calibration

Fossil calibrations were based on age ranges, with calibration intervals defined by minimum and maximum age constraints (Table 1). Minimum bounds were determined from the fossil record presented here, while maximum bounds were derived from time‐calibrated phylogenies, primarily from Rabosky et al. (2018) and Chang et al. (2019), and based on the maximum age of the 95% confidence interval for node ages. For example, in Anostomidae, the minimum age supported by the fossil record is 5.3 Ma, whereas phylogenetic analyses suggest a maximum age of up to 49 Ma. Therefore, the calibration point for the most recent common ancestor (MRCA) of Anostomidae was defined by the interval 5.3–49 Ma. Notably, no age constraint was applied to the root of the tree.

**TABLE 1:** Calibration information. For each node concerned, nodes age estimates from the present study (Estimated age), as well as minimum and maximum ages used for calibration are provided including main information on the fossil reference for the minimum age (Lundberg 1975; Langeani 1998; Malabarba 1998; Dutheil et al. 1999; Harrison et al. 2001; Malabarba and Lundberg 2007; Longbottom 2010; Chen and Chang 2011; Weiss et al. 2012; Cione and Azpelicueta 2013; Benton et al. 2015; Chen et al. 2015; Antoine et al. 2016, 2021; Mirande 2019; del Papa et al. 2022; Liu et al. 2023; Gil‐Delgado et al. 2023). Maximum age corresponds to the maximum age of the 95% confidence interval calculated in Rabosky et al. (2018). Complementary information of fossil calibration for minimum age if available in Materials and Methods S1.

Node	Clade definition in the tree	Order	Estimated age (Ma)	Maximum age (Ma)	Minimum age (Ma)	Fossil taxa	Systematic attribution main references	ICS stage	Datation main references
*1. Chanoidei*			**126.3**	**155.7**	**126.3**				
Stem Chanidae	Chanoidei (Chanidae + Kneriidae)	Gonorhynchiformes			126.3	†*Rubiesichthys gregalis*	Benton et al. (2015)	Barremian (Lower Cretaceous)	Gil‐Delgado et al. (2023)
*2. Cyprinidae*			**41.3**	**97.5**	**23.03**				
Crown Cypriniformes	All the species of the order	Cypriniformes			49.0	†*Jianghanichthys sanshuiensis*	Liu et al. (2023)	Danian to Ypresian (Paleocene to Eocene)	Liu et al. (2023)
Stem Cyprinidae	All the species of the family	Cypriniformes			34	†*Cyprinus maomingensis*	Chen et al. (2015)	Bartonian (Eocene)	Chen et al. (2015)
Stem Cyprinidae	All the species of the family	Cypriniformes			23.03	†*Huashancyprinus robustispinus*	Chen and Chang (2011)	Chattian (Oligocene)	Chen and Chang (2011)
*3. Loricaroidei*			**34**	**99.3**	**24.5**				
Crown Callichthyidae	All the species of the family	Siluriformes (Loricarioidei)			54.5	†*Corydoras revelatus*	Benton et al. (2015)	Ypresian (Eocene)	del Papa et al. (2022)
Crown Callichthyidae	All the species of the family	Siluriformes (Loricarioidei)			24.5	†*Taubateia paraiba*	Malbaraba & Lundberg et al. (2007)	Chattian (Oligocene)	Malbaraba & Lundberg et al. (2007)
*4. Siluroidei*			**45**	**123.1**	**45.7**				
Crown Siluroidei	31 families of this suborder	Siluriformes (Siluroidei)			66.0	†nov. gen. not named	Otero et al. (in press)	Maastrichtian (Upper Cretaceous)	Otero et al. (in press)
Crown Siluroidei	31 families of this suborder	Siluriformes (Siluroidei)			59.36	†Astephus sp.	Lundberg (1975)	Selandian (Paleocene)	Lundberg (1975)
“Big Africa” clade (sensu Sullivan et al. 2006)	All the species of the clade	Siluriformes (Siluroidei)			56.0	†*Nigerium wurnoense*	Otero (in press)	Thanetian to Ypresian (Paleocene)	*in* Longbottom (2010)
Stem Claroteidae	Claroteidae	Siluriformes (Siluroidei)			45.7	†*Chrysichthys mahengeensis*	Otero (in press)	Lutetian (Eocene)	Harrison et al. (2001)
*5. Characiphysi*			**93.9**	**143**	**93.9**				
Stem Charciphysi	Characiformes, Siluriformes, Gymnotiformes				93.9	Characiformes indet.	Dutheil et al. (1999)	Cenomaniam to Turonian (Upper Cretaceous)	Benton et al. (2015)
*6. Anostomidae*			**17**	**49**	**5.3**				
Crown Anostomidae	All the species of the family	Characiformes			41	†*Leporinus* sp.	Antoine et al. (2016, 2021)	Lower Barracan (Upper Middle Eocene)	Antoine et al. (2016)
Crown Anostomidae	All the species of the family	Characiformes			5.3	Anostomidae indet.	Antoine et al. (2016, 2021)	Zanclean (Pliocene)	Antoine et al. (2016)
*7. Serrasalmidae*			**20.1**	**88**	**5.3**				
Crown Serrasalmidae	All the species of the family	Characiformes			41	Serrasalminae indet.	Antoine et al. (2016, 2021)	Lower Barracan (Upper Middle Eocene)	Antoine et al. (2016)
Crown Serrasalmidae	All the species of the family	Characiformes			5.3	†*Serrasalmus* sp.	Antoine et al. (2016, 2021)	Zanclean (Pliocene)	Antoine et al. (2016)
*8. Cynodontidae*			**14.8**	**49**	**5.3**				
Crown Cynodontidae	All the species of the family	Characiformes			41	†*Hydrolycus* sp.	Antoine et al. (2021)	Lower Barracan (Upper Middle Eocene)	Antoine et al. (2021)
Crown Cynodontidae	All the species of the family	Characiformes			5.3	†*Hydrolycus* sp.	Antoine et al. (2016)	Zanclean (Pliocene)	Antoine et al. (2016)
*9. Bryconidae*			**59.3**	**63.1**	**7.3**				
Crown Bryconidae	All the species of the family	Characiformes			27.3	†*Brycon avus*	Mirande (2019)	Priabonian or Rupelian (Upper Eocene or Lower Oligocene)	Weiss et al. (2012)
Crown Bryconidae	All the species of the family	Characiformes			7.3	†*Salminus noriegai*	Cione and Azpelicueta (2013)	Tortonian (Miocene)	Cione and Azpelicueta (2013)
*10. Triportheidae*			**50.8**	**91.06**	**24.5**				
Crown Triportheidae	All the species of the family	Characiformes			24.5	†*Lignobrycon ligniticus*	Malabarba (1998)	Chattian (Oligocene) Deseadan SALMA	Malabarba (1998)
*11. Erythrinidae*			**27.3**	**29**	**21**				
Crown Erythrinidae	All the species of the family	Characiformes			24.5	Erythrinidae indet.	Antoine et al. (2016)	Chattian (Oligocene)	Antoine et al. (2016)

^†^ Indicates “fossil taxa”.

Fossil species were selected based on the following criteria: (1) their relevance for dating the evolutionary history of Characiformes within Otophysi, (2) accessibility of the specimens, and (3) robustness of their systematic attribution—ideally supported by phylogenetic studies, or at least unambiguous characters. Regarding this third criterion, identifications of characiform fishes based on dental morphology are accepted only when the fossil record is both spatially and temporally coherent (i.e., without significant gaps), allowing for the reasonable exclusion of morphological convergence with non‐characiform fishes that also exhibit marked heterodonty. Similarly, catfish spine morphology is not considered sufficient for family‐level attribution when the fossil record is too fragmentary. For each selected species or species group, the minimum known age of the stratigraphic level yielding the holotype specimen is used. For levels dated biostratigraphically, the numerical age of the upper boundary of the corresponding stage is applied, in accordance with the most recent version of the International Chronostratigraphic Chart (v2024/12; Cohen et al. (2013), updated). For radiometrically dated levels, the reported minimum age is used. Details regarding systematic attribution and dating justifications are provided in the Data S1.

### 2.4 Time Tree Estimations Among Otophysan Fishes

Divergence time was estimated using two algorithms including the Bayesian approach implemented in BEAST 2.6.7 (Bouckaert et al. 2014), and the penalized likelihood approach (Sanderson 2002) as implemented in the “chronos” function in the R package “ape” (Paradis and Schliep 2019). For the Bayesian analyses, inferences were conducted using the 20 partitions previously defined and the substitution models selected by ModelFinder were used along with the maximum likelihood (ML) estimates of their parameters, with no further parameter estimation during the analysis. Inferences were based on the Yule model (uniform birth rate) and a relaxed clock with a log‐normal distribution. The Monte Carlo Markov Chains (MCMC) were initiated with a 0.3% divergence per million years (Myrs) for rRNA and tRNA (Orti 1997; Sullivan et al. 2006) and 1.2% per Myrs for protein‐coding regions (Bermingham et al. 1997). Two MCMC runs of 200 million generations (burn‐in of 10%) were considered to check for convergence and to check ESS estimates using Tracer 1.7.1 (Drummond et al. 2012). In parallel, time‐calibrated phylogenetic reconstructions were conducted with the penalized likelihood approach implemented in the function “chronos” in the R package “ape” (Paradis and Schliep 2019). In this model, each branch has its own substitution rate but the correlation between substitution rates among adjacent branches is determined by the smoothing parameter (*λ*); the higher the *λ*, the stronger the correlation (Sanderson 2002). Three different values of *λ* were tested including 1, 10 and 100 and the performance of three different models was compared, including relaxed, correlated, and discrete clock models. The resulting nine combinations of the three *λ* values and the three clock models were compared and the best model was selected using the ɸ_IC_ criteria (Paradis 2013).

### 2.5 Ancestral Areas Estimations

With the objective to reconstruct ancestral areas along reconstructed nodes in the Ostariophysan phylogeny, the presence and absence of ostariophysan families were compiled for two sets of geographical partitions following the methodology proposed in (Sholihah, Delrieu‐Trottin, Condamine, et al. 2021; Sholihah, Delrieu‐Trottin, Sukmono, et al. 2021), and corresponding to: (1) super continental scale (i.e., Gondwana vs. Laurasia), and (2) continental scale (i.e., Africa, North and South America and Eurasia). The distribution of the species included in the present phylogenetic reconstructions were first recorded (Table S1), and the distribution of ostariophysan families were further refined by browsing country records in Fishbase (Froese and Pauly 2023). For each continent, species lists were browsed for several large and geographically representative countries to record the presence of ostariophysan families. In Eurasia for instance, checklists of the freshwater fishes from Russia, China and Indonesia were examined. Once the distribution of each family was established across the four continents considered here, the distribution in Gondwana and Laurasia was deduced according to the geological history. India was considered as belonging to Eurasia but included in Gondwana for super‐continental reconstructions.

The relative contribution of dispersal and vicariance in shaping the distribution of ostariophysan fishes was estimated using the model‐based approach of ancestral area estimation of Matzke (2014) as implemented in RASP v.4 (Yu et al. 2020). These analyses were conducted on a time tree at the family level which was obtained by pruning the tree to keep a single leaf for each family. When a terminal leaf encompassed more than a single geographic partition, multi‐regional coding was applied. Non‐otophysans (i.e., *Chanos chanos* , *Gonorhynchus greyi*) and secondary marine otophysan species (i.e., catfish family Ariidae) were removed to avoid bias during the reconstructions. Inferences of the ancestral areas were conducted using six different biogeographic models including Bayesian biogeographical inference BAYAREALIKE (Landis et al. 2013), dispersal extinction cladogenesis DEC (Ree and Smith 2008), dispersal‐vicariance analysis DIVA (Ronquist 1997), and the three alternative models including the jump dispersal parameters (BAYAREALIKE+J, DEC+J, and DIVA+J), which enable the establishment of a new lineage outside the distribution range of its ancestor through founding speciation (Matzke 2013). The inclusion of the parameter J has been recently criticized from a conceptual and statistical point of view (Ree and Sanmartín 2018). This model of founding speciation by jump dispersal has been developed specifically for island systems to account for the establishment of a new lineage without an intermediary widespread ancestor (Clark et al. 2008). Regarding the continental biogeographical scenario considered here, jumping dispersal cannot be discarded from a conceptual perspective considering opportunities for jump dispersal (e.g., India). Models of ancestral range estimation including the J parameter were thus considered here and the best‐fit model was estimated using the Akaike weight (AICc_wt) criterion.

## 3 Results

### 3.1 Phylogenetic Reconstructions

A total of 239 new mitogenomes of Characiformes were successfully assembled including 144 from the SRA files of the study by Melo et al. (2022) and 95 from the new sequencing achieved for this study (Table S1). Illumina sequencing yielded a number of reads ranging between 6 and 12 million per genomic libraries. Mitogenomes assembled from the new sequencing done here were all above 16,000 bp and assembled with a minimum coverage of 10× while 144 mitochondrial scaffolds of the 293 SRA files from Melo et al. (2022) were more than 12,000 bp long with a minimum coverage of 10×. These new mitogenomes ranged between 11,986 bp (*Hyphessobrycon* sp.) and 18,650 bp ( *Moenkhausia oligolepis* ), with an average of 16,600 bp. The gene order and composition were typical of vertebrates with 13 protein‐coding genes, two ribosomal genes, 22 tRNA, and a control region (Miya and Nishida 2015). Excepting ND6 and 8 tRNA located in the light strand, all remaining genes and tRNA are located in the heavy strand. A gene rearrangement was detected in several species belonging to the family Characidae and corresponding to a duplication of the control region and the tRNA‐Met, and the relocation of tRNAs Met, Ile, Gln and Pro between the portions coding for ND2 and COI. These rearrangements, consistent with previous observations in *Moenkhausia* (Xu et al. 2021), were found in *Moenkhausia oligolepis* and *Bario steindachnerina* and account for the unusually long mitochondrial genomes of these two species. Finally, an additional set of 77 mitogenomes of Characiformes was retrieved from GenBank.

A total of 453 mitochondrial genomes were aligned, including 316 Characiformes. The raw alignment of these 453 mitochondrial genomes with MUSCLE was 30,198 bp long, which once filtered for gene rearrangement and single large insertions was reduced to 17,466 bp including 13,539 variable sites (Table S2). Finally four genes out of the 21 from the study of Rabosky et al. (2018) presented a taxonomic coverage above 20% and were included. These include myh6, rag1, rag2 and sh3px for 324 species and corresponded to 4235 bp including 2570 variable sites (Table S2). When combined, sequences of 687 Ostariophysan species were included in the analyses for a total of 21,701 aligned positions including 15,707 variable sites (Table S2). Mitochondrial sequences were missing for 234 species, and 414, 411, 363 and 622 sequences were missing for myh6, rag1, rag2 and sh3px, respectively. Missing genes were coded as missing data (“?”) in the concatenated alignment.

**FIGURE 2:**
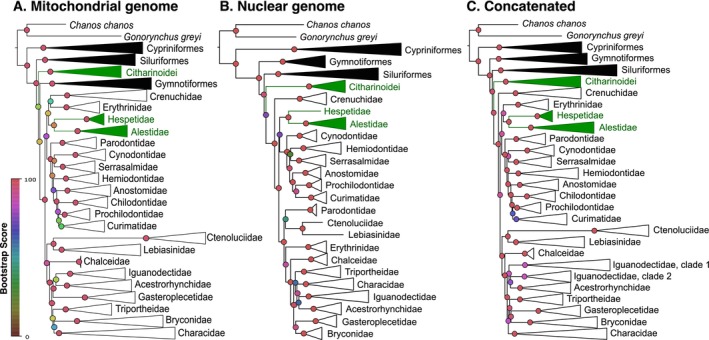
Phylogenetic reconstructions among the main Ostariophysan orders and Characiformes families. (A) reconstructions based on the 17,466 bp (i.e., 13,539 variable sites) for 529 mitochondrial genomes; (B) reconstructions based on the 4235 bp (i.e., 2570 variable sites) of nuclear genes (myh6, rag 1, rag 2, sh3px) for 324 species; (C) reconstructions based on the 21,701 aligned positions (i.e., 15,707 variable sites) of the concatenated data set for 687 Ostariophysan species.

When analyzed independently, both the mitochondrial and nuclear data sets supported the monophyly of Otophysi (Cypriniformes, Siluriformes, Gymnotiformes, Characiformes) and of Characiphysi (Siluriformes, Gymnotiformes, Characiformes) in ML analyses (Table 2; Figures S1 and S2; Trees S1 and S2; Table S3). However, mitochondrial and nuclear phylogenetic reconstructions mainly differed in the deep relationships within Characiphysi (Figure 2; Figures S1 and S2; Trees S1 and S2). Characiformes were not monophyletic with the mitochondrial data set with the Citharinoidei (Citharinidae, Distichondontidae) placed in a sister relationship as follows (Siluriformes, (Citharinoidei, (Gymnotiformes, Characoidei))). By contrast, Characiformes were monophyletic in the nuclear reconstructions and more closely related to Siluriformes as follows (Gymnotiformes, (Siluriformes, Characiformes)); however, their monophyly was moderately supported (Table 2). Within Characiphysi, deep relationships were poorly supported in ML mitochondrial reconstructions. By contrast, all deep nodes in Characiphysi were highly supported in the nuclear reconstructions, excepting the node involving the Citharinoidei, which was moderately supported (Figure 2, Table 2). Within Characiformes, the two main clades observed by Betancur et al. (2018) and Melo et al. (2022): Clade I (Alestidae, Hepsetidae, Erythrinidae, Cynodontidae, Hemiodontidae, Serrasalmidae, Parodontidae, Prochilodontidae, Chilodontidae, Curimatidae, Anostomidae) and Clade II (Ctenoluciidae, Lebiasinidae, Chalceidae, Bryconidae, Iguanodectidae, Acestrorhynchidae, Gasteropelecidae, Triportheidae, Characidae) were observed in the mitochondrial and nuclear reconstructions, excepting (i) the position of the Crenuchidae in the mitochondrial data set which were placed within Clade I next to the Erythrinidae in a poorly supported relationship and (ii) the position of the Parodontidae and Erythrinidae in the nuclear data set, i.e., they were placed in a poorly supported relationship within Clade II, respectively with the families Ctenoluciidae and Lebiasinidae, and as sister taxa of remaining species of Clade II (Figure 2 and Table 2). The concatenated data set including mitochondrial and nuclear sequences yielded a highly supported topology with deep phylogenetic relationships as follows (Gymnotiformes, (Siluriformes, Characiformes)) and the monophyly of Characiformes is highly supported (Figure 2; Table 2; Figure S3; Tree S3). Within Characiformes, Crenuchidae are placed in a basal position with high statistical support and both Clade I and Clade II from previous studies are well supported.

**TABLE 2:** The bootstrap scores following 5000 replicates for mitochondrial, nuclear and concatenated data sets among selected nodes.

	Bootstrap scores		
	mtDNA	nDNA	Concatened DNA
Taxon			
Otophysii	100	100	100
Characiphysi	100	100	100
Siluriformes + Characiformes	*	95	99
Characiformes	*	76	99
Citharinoidei	100	100	100
Characoidei	100	100	100
Characoidei clade I	*	*	95
Characoidei clade II	98	*	100
Average in Characoidei clade I	*	*	97
Average in Characoidei clade II	94	*	97

* Node not observed in phylogenetic reconstructions.

### 3.2 Time Tree Estimates

**FIGURE 3:**
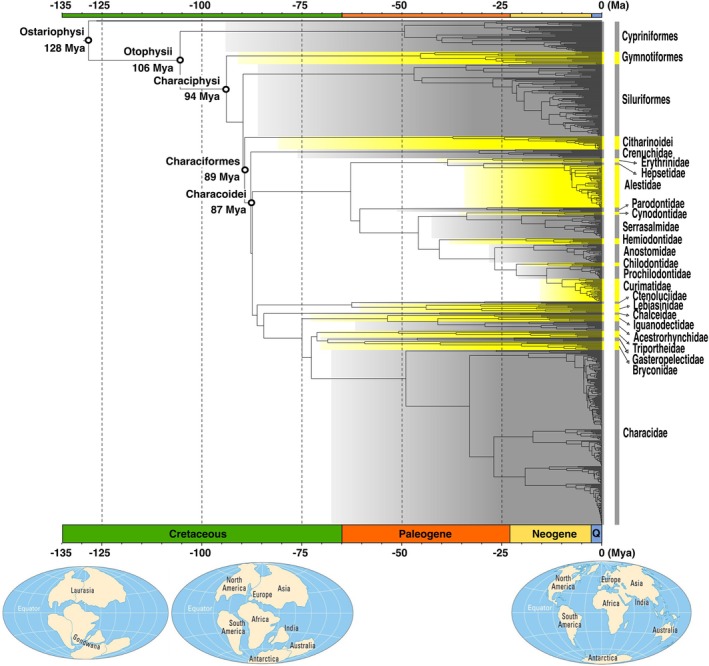
Time tree estimated using 11 fossil calibrations (Table 1) with a discrete model and *λ* = 10. The nodes associated to the origin of Ostariophysi (128 Ma), Otophysii (106 Ma), Characiphysi (94 Ma), Characiformes (89 Ma) and Characoidei (87 Ma) are highlighted with black circles.

Bayesian inferences yielded poor results, with low effective sample size (ESS) values. A single MCMC chain of 200 million required ca. 30 days to complete on a machine with a 2.3 GHz processor and 24 cores. In this context, the Bayesian approach was ultimately discarded due to its poor performance on this dataset. According to the penalized maximum likelihood approach and the ɸ_IC_ criteria, the time tree estimation based on a discrete model and *λ* = 10 was the best suited for the ML tree reconstructed using the concatenated data sets with ɸ_IC_ = 1848.89 (Table 3). This model also provided the most realistic age estimates for the root of the Ostariophysi with 128.39 Ma (Table 3). The relaxed clock model provided the most unrealistic estimates with a root of the Ostariophysi dated between 767 and 2595 Ma depending on the *λ* used. The time tree reconstructed with a discrete model and *λ* = 10 dated the root of Otophysi to 105 Ma (Figure 3; Figure S4; Tree S4). The respective ages of the crown groups ranged 94–37 Ma with Characiphysi (93.9 Ma), Gymnotiformes (45.35 Ma), Siluriformes (46.97 Ma), and Characiformes (89.35 Ma) (Figure 3). Within the Characoidei, the divergence between the African lineage Alestoidea (Alestidae + Hepsetidae) from its South American relatives was estimated to be 29.57 Ma. This drastically contrasts with the divergence between the African lineage Citharinoidei and the South American Characoidei dated around 87.79 Ma.

**TABLE 3:** Summary statistics of the penalized likelihood procedure implemented to reconstruct a dated time tree among Ostariophysan families including the correlated, relaxed and discrete models with their ɸ_IC_ score and estimated ages for the roots of ostariophysan and characiformes.

	Smoothing parameter lambda (*λ*)			
		*λ* = 1	*λ* = 10	*λ* = 100
Models	Correlated	ɸIC = 4518.35	ɸIC = 4516.63	ɸIC = 4516.63
		Root dating = 466 My Characiform dating = 114 My	Root dating = 465 My Characiform dating = 100 My	Root dating = 462 My Characiform dating = 132 My
	Relaxed	ɸIC = 4517	ɸIC = 4517.76	ɸIC = 4520.1
		Root dating = 767 My Characiform dating = 137 My	Root dating = 953 My Characiform dating = 93 My	Root dating = 2595 My Characiform dating = 93 My
	Discrete	ɸIC = 2e+100	ɸIC = 1848.89	ɸIC = 2e+100
		Root dating = 133 My Characiform dating = 124 My	Root dating = 128 My Characiform dating = 89 My	Root dating = 203 My Characiform dating = 104 My

The early diversification of the Otophysi likely started around 105 Ma with the initial split of Cypriniformes and Characiphysi. This divergence predates the last stages of the separation between Africa and South America (estimated around 85 Ma; Scotese et al. 2025). However, the early diversification of the Characiphysi crown group is estimated to be concomitant with Western Gondwanan fragmentation (93.9 Ma). The divergence of the two Characiformes suborders (Citharinoidei and Characoidei) is thought to have occurred simultaneously with these events (89.35 Ma), but with 29.57 Ma, the age of the African Characoidei clade (i.e., Alestoidea) largely postdates this ancient vicariance event and the full opening of the Atlantic Ocean (Aminov et al. 2023).

### 3.3 Ancestral Areas Estimations

**FIGURE 4:**
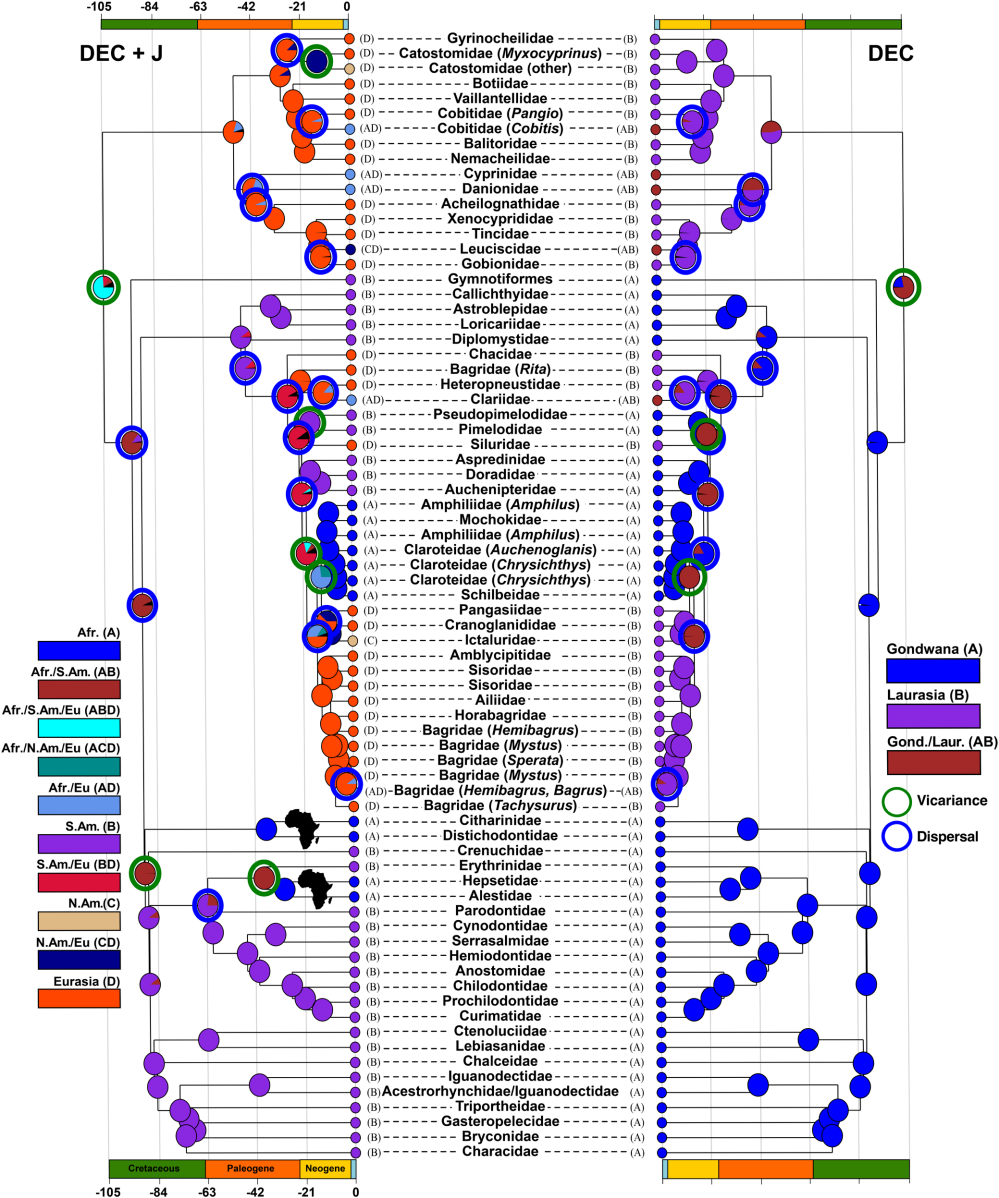
Ancestral areas estimates according to DEC+J for a partitioning per continents (South America, Africa, North America and Eurasia) and DEC for a partitioning according to the supercontinent (Laurasia and Gondwana). Major inferred vicariance events are highlighted in green, and major dispersal events are highlighted in blue.

The DEC model presents the best AICc weight in the two sets of spatial partitions analyzed (AICc_wt, Table 4). However, the inclusion of the J parameter clearly improves the AICc weight for the continental partitioning. Based on the two partition schemes, the most likely scenario includes the following series of events (Figure 4). First, the inferred ancestral range of the Otophysi spread over a wide area including Africa, South America and Eurasia, and corresponding to Gondwana and Laurasia. Second, the basal divergence between Cypriniformes and Characiphysi is inferred to result from a vicariance event between Eurasia (i.e., Laurasia) and Africa and South America (i.e., Gondwana). Third, the divergence between Gymnotiformes and Siluriformes is inferred to result from a reorganization of their range corresponding to a contraction to South America within Western Gondwana as dispersal events are reconstructed for these nodes (Figure 4). Finally, African Characiformes are inferred to originate from two distinct vicariance events at 89.35 Ma and 29.57 Ma: (1) the Citharinoidei are inferred to originate from a vicariance event which matches the age estimate of the isolation of Africa and South America (around 89 Ma, Figure 4); (2) the African Characoidei lineages (i.e., Alestoidea clade) are inferred to originate from an expansion of their range from South America to Africa, between 40 Ma and 60 Ma, followed by a vicariance event around 29 Ma, thus widely post‐dating the separation of the landmasses.

**TABLE 4:** Summary statistics of the ancestral area model estimates as implemented in Rasp with corrected Akaike Information Criterion (AICc), their weight (AICc_wt), associated *p*‐value and inclusion of the J parameter.

Time scale	Biogeographic model	AICc	AICc_wt	*p*val	+J parameter
Current	DEC	47.86	0.022	0.0016	Yes
	DEC+J	40.31	0.97		
	DIVA	51.03	0.0045	0.11	Yes
	DIVA +J	50.91	0.0048		
	BAYAREALIKE	66.44	2.10E‐06	0.0001	Yes
	BAYAREALIKE+J	54.14	0.001		
Ancestral	DEC	11.18	0.71	1	No
	DEC+J	13.23	0.25		
	DIVA	17.88	0.025	1	No
	DIVA +J	19.91	0.009		
	BAYAREALIKE	28.39	0.0001	0.0049	Yes
	BAYAREALIKE+J	22.48	0.0025		

## 4 Discussion

Intensively debated for decades, phylogenetic reconstructions among otophysan orders have significantly progressed during the last decade with the use of dense taxonomic sampling and ample genomic resources, resulting in revised classifications of several groups (Betancur et al. 2018; Tan and Armbruster 2018). The present study confirms the difficulties in reliably reconstructing deep phylogenetic relationships within Characiformes, with the Citharinoidei being part of Characiformes (Fink and Fink 1981; Dimmick and Larson 1996; Lavoué et al. 2005; Arcila et al. 2017; Betancur et al. 2018; Rabosky et al. 2018) or placed in a sister relationship with other otophysan orders (Nakatani et al. 2011; Chen et al. 2013; Dai et al. 2018; Melo et al. 2022). Although previous studies leveraged extensive taxonomic sampling to investigate phylogenetic relationships within Citharinoidei, the placement of the group among other ostariophysan lineages has remained largely unresolved (Arroyave et al. 2013, 2020). Alternatively, studies focusing on phylogenetic relationships within Characiformes and questioning their monophyly were based on datasets with low taxonomic coverage outside Characiformes (Chakrabarty et al. 2017; Melo et al. 2022). Here, leveraging a dense taxonomic sampling of African lineages for a large data set combining mitochondrial and nuclear genes provided a robust phylogenetic reconstruction of Characiformes intra‐relationships supporting the monophyly of the order. Besides, several new and highly supported relationships within Characiformes such as the position of the African super‐family Alestoidea close to the South American Erythrinoidea suggest an artefactual position of several lineages in previous phylogenomic studies (Betancur et al. 2018; Melo et al. 2022). However, Melo and Stiassny (Melo and Stiassny 2024) recently demonstrated that the African families Alestidae and Hepsetidae are more closely related to Erythrinoidea and Curimatoidea within Characoidei than to Citharinoidei—a result that is consistent with the present phylogenetic reconstructions.

### 4.1 Early Branching in the Otophysan Tree of Life

Individual reconstructions based on mitochondrial genomes or nuclear genes resulted in congruent relationships at the root of the ostariophysan and otophysan clades as the topology (Gonorhynchiformes, (Cypriniformes, Characiphysi)) was recovered with high statistical support with both genomic compartments (Figure 2). This topology was also observed in previous molecular and morphological studies (Fink and Fink 1981; Nakatani et al. 2011; Chen et al. 2013; Betancur et al. 2017) and is now widely accepted. As expected, the relationships between Gymnotiformes, Siluriformes, Citharinoidei and Characoidei were more conflictual and an apparent disagreement between mitochondrial sequences, supporting the topology (Siluriformes, (Citharinoidei, (Gymnotiformes, Characoidei))), and nuclear sequences supporting (Gymnotiformes, (Siluriformes, (Citharinoidei, Characoidei))). The topology recovered within Characiphysi with the mitochondrial data set alone, however, was poorly supported with very short internal branches suggesting a lack of informative characters in support of the mitochondrial topology. This hypothesis was confirmed by the improvement of the statistical support in individual and concatenated data sets with a significant increase of the bootstrap scores for the monophyly of Characiformes (i.e., not observed in mitochondrial data, 76 for nuclear genes and 99 for the concatenated data set), the sister relationships between Siluriformes and Characiformes (i.e., not observed with mitochondrial data, 95 for nuclear genes and 99 for the concatenated data set), Characoidei clade I (i.e., not observed in mitochondrial and nuclear reconstructions but supported by a bootstrap score of 99 for the concatenated data set) and Characoidei clade II (i.e., not observed in nuclear reconstructions but supported by a bootstrap score of 98 and 100 for the mitochondrial and concatenated data set, respectively) (Table 2). Thus, these apparent discrepancies are more reflective of a lack of information in individual data sets in fast evolving clades (i.e., soft polytomy) than conflicts in the evolutionary signal (e.g., hard polytomy) as combining data sets improved the statistical support of the reconstructions (Walsh et al. 1999; Slowinski 2001). Besides, molecular saturation in the mitochondrial genomes was previously observed at the root of the Characiformes due to explosive radiation during their early diversification (Orti and Meyer 1997; Calcagnotto et al. 2005; Hubert et al. 2005). Considering that the phylogenetic signal is additive while the random noise is average (Wenzel and Siddall 1999), the increase in the statistical support observed here is unlikely to result from known artifacts such as the integration of paralogs, incomplete lineage sorting or introgressive hybridization during the early stage of diversification (Takahashi et al. 2001; Suh 2016; Ford et al. 2019; Alda et al. 2021).

The monophyly of Characiformes, highly supported here, is further supported by multiple anatomical synapomorphies (Fink and Fink 1981). However our study does not support the sister relationships between Siluriformes and Gymnotiformes, supported by morphology‐based reconstructions (Fink and Fink 1981) and some molecular studies (Arcila et al. 2017; Hughes et al. 2018). Instead, a relationship between Siluriformes and Characiformes (Characoidei + Citharinoidei) is reconstructed in agreement with several molecular studies (Chen et al. 2013; Rabosky et al. 2018). These deep relationships are mainly supported by nuclear genes, which also confidently resolved interfamilial relationships within Characiformes, as the superfamilies defined by Betancur et al. (2018) are monophyletic in the nuclear reconstructions. These results provide a robust phylogenetic framework highlighting the importance of the nuclear genome in resolving deep relationships, and that of the mitochondrial genome in resolving more recent interfamilial relationships. Our study confirms the benefits of combining sequences from genomes with distinct evolutionary histories and evolutionary rates as the additive signal carried by each genomic compartment might be expected to provide robust reconstructions. However, attention should be paid in combining data sets with partially overlapping data as sets of sequences that are not concatenable among markers may lead to artefactual paraphyly/polyphyly. The family Iguanodectidae for instance is monophyletic for both mitochondrial and nuclear sequences separately, in a sister relationship with Acestrorhynchidae. However, two distinct clades are reconstructed in the concatenated data set with clade 1 corresponding to mitochondrial genomes with nuclear genes coded as missing data and vice versa in clade 2.

### 4.2 Timeframe of the Diversification of Characiphysi

Calibrating phylogenetic reconstructions for dating cladogenetic events is a challenging task for such ancient and diverse groups, and incongruent estimates are not uncommon depending on the methods of calibration used (Inoue et al. 2009). Our calibration based on multiple fossil records distributed across all ostariophysan lineages provided a dated time tree with age estimates compatible with several previous studies, fossil records and major geological events during the fragmentation of Pangaea (Chen et al. 2013; Betancur et al. 2017; Capobianco and Friedman 2018; Dai et al. 2018; Aminov et al. 2023; Scotese et al. 2025). However, our age estimates for the deeper divergences are on the youngest edge of previous estimates with the MRCA of Characiphysi dated around 94 Ma here versus 225 to 106 Ma in previous studies, and the MRCA of Gymnotiformes dated around 45 Ma here versus 180 to 70 Ma previously (Arroyave et al. 2013; Chen et al. 2013; Betancur et al. 2017; Melo et al. 2022; Melo and Stiassny 2024). The MRCA of Siluriformes is surprisingly younger with an age estimated around 47 Ma, an estimate substantially younger than previous estimates with an age ranging 175–82 Ma (Nakatani et al. 2011; Chen et al. 2013; Dai et al. 2018; Melo et al. 2022). These differences might be attributed to the absence of ancient lineages in our dataset; however, given the dense taxonomic sampling employed in this study, this explanation appears unlikely at least for Characiformes. Alternatively, differences in topology may account for the differences observed in age estimates between the present studies and previous estimates. For instance, we place the origin of Characiformes between 90 and 89 Ma, an estimate consistent with the majority of previous studies which placed their origin between 120 and 80 Ma (see Capobianco and Friedman 2018). Other studies support a much older origin; for example Nakatani et al. (2011) estimate it at 220 Ma, and Melo et al. (2022) at 160 Ma. Regardless of the fossil record or the calibration method used, these studies reconstructed the Characiformes as polyphyletic, with the Siluriformes more closely related to Characoidei than Citharinoidei. As in other studies supporting this topology, the MRCA of Characiformes is older than 120 Ma. The fossil record used here also substantially differs from the recent studies inferring a much ancient origin of Characiformes. Melo and Stiassny (2024) rely on an ancient fossil record of Characiformes, mostly mandibular elements tracing back to the Cretaceous, and assign them to several infra‐familial nodes. These calibration points are questionable considering the high homoplastic condition of these mandibular elements. For instance, Vullo et al. (2017) and Kölbl‐Ebert et al. (2018) recently published piranha‐jawed pycnodont fishes from the Cretaceous‐Paleogene of Morocco and Late Jurassic of Germany, respectively. These pycnodont fishes present a very similar mandibular structure to what is observed in Serrasalmidae (piranhas), whose origin is consistently estimated to occur during the late Neogene by molecular phylogenetic studies (Hubert et al. 2007; Kolmann et al. 2021).

### 4.3 Pangean Vicariance, Gondwanian Diversification and Trans‐Continental Dispersal

Our estimated ancestral ranges are consistent with a Pangean origin of the ancestor of otophysi fishes with a divergence due to a vicariance between Cypriniformes, which are inferred to originate from Laurasia and Characiphysi, whose origin is positioned in Gondwana (Figure 4). This result is in agreement with the model of divergence by vicariance through continental drift during the weakening of Pangea (Diogo 2004). However, our estimate of the age of this vicariance event is younger than the geological estimates which place the separation of Laurasia and Gondwana around 140 Ma (Chen et al. 2013). This delay between the fragmentation of the Pangea landmass and the emergence of a distinct Cypriniformes lineage to the North and a distinct Characiphysi lineage to the South was however expected. Otophysan extinctions were previously reported in North America and Europe in the Cretaceous as characiform‐like fossils were reported in North America and Europe during the Late Cretaceous global warming (Grigorescu et al. 1985; Otero et al. 2008; Newbrey et al. 2009; Brinkman et al. 2013; Gaudant 2014). The extinction of these widely distributed otophysan lineages during the separation of Laurasia and Gondwana suggests the co‐existence of fossil stem groups in the fragmenting Pangea until a subsequent faunal replacement resulted in the segregation of each group in Laurasia and Gondwana (Chen et al. 2013). The end of the green house during the Cretaceous with an extended tropical belt at higher latitude (Ziegler et al. 2003; Morley 2011) likely accounts for the faunal replacement in Laurasia and Gondwana and for the delay in the establishment of reciprocally monophyletic Cypriniformes and Characiphysi in Laurasia and Gondwana respectively. Alternatively, a secondary colonization of Gondwanan freshwater systems by a marine ancestor is highly unlikely, as no marine otophysans are currently known. The only extant otophysan lineage capable of tolerating saltwater is the catfish family Ariidae. Moreover, all otophysan fossils described to date are exclusively associated with freshwater environments (Lundberg 1998; Lundberg et al. 1998).

Our biogeographic reconstructions place the origin of modern Characiphysi in Gondwana, with an ancestral range estimate extending across South America and Africa; however, the restriction of Gymnotiformes and early siluriform lineages to South America is inferred to result from a contraction of their ancestral range within Gondwana, with the MRCA of Characiphysi being inferred to occur across Gondwana (Figure 4). Besides, BIOGEOBEARS‐based reconstructions excluded major extinction or vicariance events among the early diversifying lineages of Characiphysi suggesting that reshuffling is more likely as a consequence of range rearrangement through dispersal. However, this hypothesis contradicts that emphasized by Diogo (2004) and Briggs (2005) and suggests that these lineages originated from vicariance events that preceded or were the consequence of the separation of South America and Africa and subsequent extinctions. Our reconstructions are consistent with major range rearrangement at the end of the Cretaceous, which occurred in Laurasia, but also apparently in the tropical belt within Gondwana as well.

Ancestral range estimates within Characiformes point to distinct mechanisms at the origin of the African lineages. Two competing hypotheses have been previously proposed to account for their transatlantic distribution; the first hypothesis relies on a vicariance event within Gondwana (Novacek and Marshall 1976), and the second implies range rearrangements after the fragmentation of Gondwana and the separation of Africa and South America (Briggs 1979). Competing hypotheses of phylogenetic relationships among African and South American lineages of Characiformes have largely contributed to perpetuating the debate about their origin. Recent phylogenomic analyses based on UCE or exons‐capture resulted in the placement of African lineages at the root of Characiformes, supporting an origin by vicariance associated with the separation of Gondwana and Africa around 100 Ma (Betancur et al. 2018; Melo et al. 2022). Such a pattern of vicariance was observed in our reconstructions between the Citharinoidei and the early South American Characoidei at 89 Ma (Figure 4). Although the dating of this event is often discussed (see above), there is unanimous agreement that these lineages diverged by vicariance (Chen et al. 2013; Betancur et al. 2018; Melo et al. 2022). Whatever the age estimates for the origin of Characiformes, a largely unequal level of species richness is observed between these two sub‐orders, with 118 valid species of Citharinoidei versus 2361 of Characoidei (Eschmeyer et al. 2025). This large unbalance can be explained by two hypotheses: (1) vicariance occurred between already diversified lineages, associated with the extinction of part of the African lineages (e.g., Characoidei); (2) this vicariance would have taken place between two lineages at an early stage of their diversification and the later South American clade would have diversified to a greater extent. The first hypothesis is unlikely as, to our knowledge, fossils of Characoidei are still to be discovered in Africa (Arroyave et al. 2013) and an accelerated rate of diversification has been recently detected among Characoidei during the last 30 Myr (Melo et al. 2022). These differences are likely to be explained by the differences observed in the hydrographic systems in terms of size between the Amazon and central Africa, both in terms of complexity and fragmentation, i.e., abiotic parameters playing a role in the macroevolutionary dynamics of diversification (Abell et al. 2008; Arroyave et al. 2013).

The position of the African Alestoidea in a sister relationship with Erythrinoidea is more intriguing as the topology indicates that this divergence occurred separately from other African lineages and its age estimates around 35 Ma indicate that it largely postdated the fragmentation of Gondwana and the separation of South America and Africa. Our reconstructions support a range expansion from South America to Africa across a proto‐ocean, between 65 and 35 Ma during the Upper Cretaceous–Paleocene, followed by a vicariance event between the African (Alestoidea) and South American (Erythrinidae) lineages (Figure 4). Melo and Stiassny (2024) provided significantly older estimates for the divergence between the African Alestidae and the South American Erythrinoidea and Curimatoidea, dating it to approximately 110 Ma and suggesting a distinct geological timeframe. However, the hypothesis of a vicariant origin followed by widespread extinction of Characiformes in Africa is not supported by the current fossil record. (Lundberg 1993). Two explanations are likely to explain transcontinental dispersal, which are not exclusive: (1) a paleoclimatic scenario that generated transcontinental dispersal following rearrangements of ecological niches, (2) a paleogeological scenario that allowed migrations between continental masses. A global cooling initiated during the Paleocene, after a peak of warming ca. 50 Ma, resulted in a shrinking of the tropical belt and the lowering of the world oceans (Westerhold et al. 2020). Thus, numerous eustatic, hydrobiological and geological changes occurred during the Upper Cretaceous–Paleocene that enabled transcontinental dispersal between South America and Africa, a pattern that received much support during the last decades (de Queiroz 2005; Lundberg et al. 2007; Vidal et al. 2008; de Oliveira et al. 2009; Gamble et al. 2011; Chen et al. 2013). Transcontinental dispersal is consistent with the claims made earlier by Lundberg et al. (1998), who suggested that much of the diversity of freshwater fishes could be the result of paleo‐hydrobiological changes that favored dispersal followed by divergence by vicariance. Such transmarine vertebrate migrations could be achieved by several means including: (1) the formation of ‘corridors’ compatible with the biology of the species, during strong freshwater pulses combined with low sea levels and desalination of surface waters (Lundberg et al. 2007), (2) a ‘stepping stone’ model during sporadic terrestrial connections between 60 and 40 Ma that was notably proposed for mammals (de Oliveira et al. 2009; Warren et al. 2010), or even by floating raft events (Antoine et al. 2012; Marivaux et al. 2023) or zoochory of eggs. The occurrence of long‐distance dispersal in otophysans is further supported by the significant improvement of the likelihood of BIOGEOBEARS models for continents when incorporating the jump dispersal parameter. Such expansion of the range of descendant lineages outside the range distribution of their ancestors cannot be discarded and is supported in several cases such as for Alestoidea. These possibilities can be explained by physiological and/or behavioral adaptations in the species distributed on both continents. Alestoidea for instance are mostly small and opportunistic fish species with omnivorous diets, drought‐resistant eggs and adaptations to low‐oxygen environments (Arcila et al. 2018; Ishimatsu et al. 2018; Burns 2021), various characteristics which could reflect the adaptations that enabled them to disperse, including through terrestrial connections.

## 5 Conclusions

Our study supports the efficiency of combining mitochondrial genome with nuclear gene sequences in resolving the relationships among recalcitrant groups of otophysans. The resulting topology provided reconstructions congruent with the literature for the groups whose phylogenetic relationships are now well established, while challenging others for difficult groups with new and highly supported hypotheses of relationships. Our biogeographic reconstructions are consistent with the significant impact of plate tectonics and the fragmentation of Pangea on the diversification of otophysan fishes. The separation of Pangea into Laurasia and Gondwana is inferred to be the main mechanism promoting the divergence between Cypriniformes and Characiphysi; however, more complex faunal rearrangements are foreseen in light of fossil records at the end of the warm climate of the Cretaceous. Our study indicates that transcontinental dispersal during the Upper Cretaceous–Paleocene is required to explain several events of range expansion among Characiformes and further calls for an exploration of the mechanisms that enabled such long‐distance dispersal.

## Author Contributions

**Achille Lenglin:** data curation (lead), formal analysis (equal), investigation (equal), writing – original draft (equal). **Max Hidalgo:** data curation (equal), investigation (equal), resources (equal). **Guido Miranda:** data curation (equal), investigation (equal), resources (equal). **Aaron De la Sota:** investigation (equal), resources (equal). **Pierre Caminade:** data curation (equal), formal analysis (equal), resources (equal), visualization (equal). **Khalid Belkhir:** conceptualization (equal), formal analysis (equal), methodology (equal), software (equal), supervision (equal), visualization (equal), writing – review and editing (equal). **Olga Otero:** data curation (equal), validation (equal), visualization (equal). **Pierre‐Olivier Antoine:** conceptualization (equal), formal analysis (equal), investigation (equal), methodology (equal), supervision (equal), validation (equal), visualization (equal), writing – review and editing (equal). **Carmen Garcia‐Davila:** conceptualization (equal), investigation (equal), project administration (equal), resources (equal), writing – review and editing (equal). **Nicolas Hubert:** conceptualization (equal), data curation (equal), formal analysis (equal), funding acquisition (equal), investigation (equal), methodology (equal), project administration (equal), resources (equal), supervision (equal), validation (equal), writing – original draft (equal).

## Conflicts of Interest

The authors declare no conflicts of interest.

## Supporting information


**Data S1:** Fossil record used in the present study.

**Figure S1:** Phylogenetic reconstruction based on mitochondrial genomes with bootstrap scores derived from 5000 replicates.

**Figure S2:** Phylogenetic reconstruction based on four nuclear genes with bootstrap scores derived from 5000 replicates.

**Figure S3:** Phylogenetic reconstruction based on the concatenated mitochondrial genomes and four nuclear genes with bootstrap scores derived from 5000 replicates.

**Figure S4:** Time tree reconstructed with the discrete model and *λ* = 10.

**Table S1:** The list of gene partitions used here including the number of species included, size of the partitions, sites without gaps, informative sites and variable sites.

**Table S2:** The list of taxa examined in this study, along with their ancestral and current distribution, the GenBank accession numbers of the corresponding molecular sequences, the assembly methods used for the mitogenomes and the origin of the reads. NA, North America; SA, South America.

**Table S3:** Most likely substitution model for each partitions according to MODELFINDER.

**Data S2:** Phylogenetic reconstruction based on mitochondrial genomes with bootstrap scores derived from 5000 replicates in newick format.

**Data S3:** Phylogenetic reconstruction based on four nuclear genes with bootstrap scores derived from 5000 replicates in newick format.

**Data S4:** Phylogenetic reconstruction based on the concatenated mitochondrial genomes and four nuclear genes with bootstrap scores derived from 5000 replicates in newick format.

**Data S5:** Time tree reconstructed with the discrete model and *λ* = 10 in newick format.

## Data Availability

The 239 newly generated mitochondrial genomes are available in the NCBI GenBank (PV962424—PV962665). DNA sequence alignments are available in Dryad (https://doi.org/10.5061/dryad.crjdfn3fj).
