# Supplementary material for: Early Biogeography of Otophysi Points to the Neotropics as the Cradle of Characiphysan Fishes
Source: Ecol Evol. 2025 Nov 15;15(11):e72431. doi: 10.1002/ece3.72431 (PMC12619110; doi:10.1002/ece3.72431)
Supplement: Supplementary file 1 — Data S1: Fossil record used in the present study. [file ECE3-15-e72431-s012.pdf]

Supplementary data 1. Systematic and dating justification for minimum age.

Stem Chanidae: 126.3 Ma
†*Rubiesichthys gregalis* [El Montsec, Spain]. Its phylogenetic position was discussed by Poyato-Ariza (1996) who confirmed its attribution to the family Chanidae based on morphological data. Lately, Near et al. (2014) recover this attribution based on combined molecular and morphological data. This fish was also used as calibration point by Benton et al. (2015) for the family. Latest datation of the limestones of El Montsec provides a Barremian age (125.8 to 121.4 ±0.6) (Gil-Delgado et al. 2023). We follow Rasbosky et al. (2018) with a minimum age estimate of 126.3 Ma.

Crown Cyprinidae: 23.03Ma
†*Jianghanichthys sanshuiensis* [lower and middle Buxin Formation]. Fossil cypriniforms from frequent in Eocene deposits from China (Chang and Chen 2008). In particular, Liu et al. (2024) revised †*Jianghanichthys sanshuiensis* and attributed it to an extinct family of Cypriniformes. The age of the Lower and Middle Buxin Formation is currently estimated to be Paleocene to Lower Eocene, i.e. between 66Ma and 49Ma (*ibid*.).
†*Cyprinus maomingensis* [Youganwo Formation in the Maoming Basin, Guangdong, China]. Fossil Cyprinidae dated to the Bartonian–Priabonian (late Eocene) and dated ~37–34 Ma by Chen et al. (2015).
†*Huashancyprinus robustispinus* [Ningming Formation, Guangxi, China]. Fossil attributed to the genus *Cyprinus* and dated to the Eocene (23.03 Ma) by Chen and Chang (2011).

Crown Loricaroidei: 24.5 Ma
†*Corydoras revelatus* [Mais Gordo Formation, Argentina]. This fossil, first described by Cockerell (1925) base on a single articulated specimen. We follow Lundberg et al. (2007) Benton et al. (2015) who used the species as calibration point for crown Callichthyidae. Recent works provide new constraint on the Mais Gordo Formation and a Minimum age constrained of 55 Ma for the top of the Mais Gordo Formation (Del Papa et al. 2022).
†Taubateia Paraiba [Tremembé Formation, Taubaté Basin, São Paulo, Brésil]. Fossil assigned to the family Loricaridae by Malabarba and Lundberg (2007) and dated between 20 and 30 Ma. We follow Rasbosky et al. (2018) with a minimum age estimate of 24.5 Ma.

Crown Siluroidei: 45.7 Ma
Nov. gen. not named [El Molino Formation at Hotel Cordillera]. Part of the material initially attributed to †*Rhineastes* by (Gayet 1991), actually belongs to a large Aspredinoidea fish based on nuchal shield morphology and connexions with the neurocranium (Otero et al., in press). Waiting for the formal taxon description and more precise systematic assignment, we confidentially attribute it to crown Siluroidei. Recent discussions of the younger Tiupampan mammalian level (Muizon & Ladevèze 2020) suggest that the underlying El Molino Formation is strictly Campano-Maastrichtian in age.
†*Arius fraasi* [Gebel Mokattam, Fajum, Egypt]. Various marine fossils have been placed in extinct species of Ariidae, including from Palaeocene and Lower Eocene of Angola and from Lutetian deposits in Mokattam, Egypt (Peyer 1928). The latter fossil, attributed to †*Arius fraasi*, is retained here since it displays clear evidence for an attribution to crown Ariidae (the presence of a ventral keel typical of the Ariinae, *sensu* Acero and Betancur, 2007). Mokattam Formation is Lutetian (Said 1962).
†*Nigerium wurnoense* [Iullemenden at Sokoto, Nigeria]. Longbottom (2010) attributed the genus to Claroteidae following Mo (1991) definition. Following Otero (in press), since the monophyly of Claroteidae *sensu* Mo (1991; not recovered here), †*Nigerium* is attributed to the

clade Big Africa *sensu* Sullivan et al. (2006) minimum age of the clade Big Africa (recovered here).
†*Chrysichthys mahengeensis* [Mahenge, Tanzania]. The attribution to the family Claroteidae is supported by the morphology of Weberian apparatus and the skull described in Murray and Budney (2003). However, the attribution to *Chrysichthys* is not supported (Otero in press). Harrison et al. (2001) proposed an age of 45.83 ± 0.17 Ma.

Stem Characiphysi: 93.9 Ma
Various characiformes [El Molino Formation, Bolivia]. Various characiform fish remains are recovers in lower, middle, and upper members of El Molino Formation (Gayet 1991, Gayet et al. 1998). Mostly teeth are available but also some teeth articulated on jaw (e.g. Gayet et al. 2001). El Molino formation spans across the Maastrichtian and probably starts in the Campanian so that an early record at 72.2Ma is reasonable.
Characiformes indet. [Kem Kem Bed Formation, Morocco]. Characiformes fragmentary remains not attributable to a specific group and assigned to the order by Dutheil (1999). Estimated age revised by Benton et al. (2015) and estimation of 93.9 Ma subsequently used by Rabosky et al. (2018). We follow this minimum age estimate here.

Stem Anostomidae: 5.3 Ma
†*Leporinus* sp. [e.g. CTA-29, 47, 66, Contamana, Peru]. The vertebrate localities of Amazonia show a subcontinuous fossil record of Anostomidae teeth whose morphology corresponds exactly to that of the extant species of genus *Leporinus*. The record starts with fossils from the Middle Eocene in Contamana (Antoine et al. 2016), and then from the Upper Eocene to the Upper Miocene (Antoine et al. 2021). The earliest layer with *Leporinus sp.* teeth is dated from the Barrancan with minimum age of 41 Ma (Antoine et al. 2016).
Anostomidae indet. [Shapaja Formation, San Martin, Peru]. Unassigned teeth assigned to the family by Antoine et al. (2021) with earliest occurrence at 5.3 Ma.

Crown *Serrasalmidae*: 5.3 Ma
Serrasalminae indet. [e.g. CTA-47, 51, 60, Contamana, Peru]. The vertebrate localities of Amazonia show a subcontinuous fossil record of Serrasalminae teeth whose morphology corresponds exactly to that of the extant pacu fish. The record starts with fossils attributed to Serrasalminae indet. from the Middle Eocene in Contamana (Antoine et al. 2016), and follows with fossils attributed to cf. *Colossoma* sp. from the Upper Eocene to the Upper Miocene (Antoine et al. 2021). The earliest layer with Serrasalminae indet. teeth is dated from early Barrancan with minimum age of 41 Ma (Antoine et al. 2016).
†*Serrasalmus* sp. [Shapaja Formation, San Martin, Peru]. Tricuspid teeth assigned to the genus *Serrasalmus* by Antoine et al (2021) with earliest occurrence at 5.3 Ma.

Stem Cynodontidae: 5.3 Ma
cf. *Hydrolycus* [CTA-29, Contamana, Peru]. The vertebrate localities of Amazonia show a subcontinuous fossil record of Anostomidae teeth whose morphology corresponds exactly to that of the extant species of genus *Hydrolycus*, from the Upper Eocene to the Upper Miocene (Antoine et al. 2021). The earliest layer with *Hydrolycus* sp. teeth is dated from early Barrancan with minimum age of 41 Ma (Antoine et al. 2016).
Hydrolycus sp. [Shapaja Formation, San Martin, Peru]. Monocuspid, sharp and dagger-like teeth assigned to the genus *Hydrolycus* by Antoine et al (2021) with earliest occurrence at 5.3 Ma.

Crown Bryconidae: 7.3 Ma

†*Brycon avus* [Entre-Córregos Formation, Aiuruoca basin, Brazil]. A total-evidence analysis places the genus as crown Bryconidae (Mirande, 2018). The minimum age of the fossil fish bearing level is 27 Ma (Malabarba 2004).
†*Salminus norai* [Toma Vieja Formation, Entre Ríos, Argentina]. A well-preserved, three-dimensional fossil skull, allowing confident assignment to the genus *Salminus* (Cione & Azpelicueta, 2013), dated at 7.3 Ma.

Crown Triportheidae: 24.5
†*Lignobrycon ligniticus* [Tremembé Formation, Taubaté Basin, Brazil]. The attribution was discussed by Malabarba et al. (1998). The age of the Tremembé Formation is Late Oligocene. The age estimate follows the age estimate used by Rabosky et al. (2018).

Crown Eryhtrinidae: 21
Erythrinidae sp [e.g. CA32 Pozo Formation, Contamana, Peru]. Teeth assigned to the family Erythinidae by Antoine et al (2021) with earliest occurrence at 21 Ma.

References :

Acero, P.A. and R. Betancur-R. 2007. Monophyly, affinities, and subfamilial clades of sea catfishes (Siluriformes: Ariidae). *Ichthyol. expl. Freshwaters* 18(2) 133–143.

Antoine, P.O., Yans, J., Castillo, A.A., Stutz, N., Abello, M.A., Adnet, S., Custodio, M.A., Benites-Palomino, A., Billet, G., Boivin, M., et al. 2021. Biotic community and landscape changes around the Eocene–Oligocene transition at Shapaja, Peruvian Amazonia: Regional or global drivers? Global and Planetary Change 202, 103512.

Antoine, P.O., M.A. Abello, S. Adnet, A.J.A. Sierra, Patrice Baby, G. Billet, M. Boivin, Y. Calderon, A. Candela, J. Chabain, F. Corfu, D.A. Croft, M. Ganerod, C. Jaramillo, S. Klaus, L. Marivaux, R.E. Navarrete, M.J. Orliac, F. Parra, M.E. Perez, F. Pujos, J.-C. Rage, A. Ravel, C. Robinet, M. Roddaz, J.V. Tejada-Lara, J. Velez-Juarbe, F.P. Wesselingh and R. Salas-Gismondi. 2016. A 60-million-year Cenozoic history of western Amazonian ecosystems in Contamana, eastern Peru. Gondwana Res. 31: 30–59.

Chang, M.-M., Chen, G. 2008. Fossil Cypriniformes from China and its adjacent areas and their palaeobiogeographical implications. pp. 337–350. *In* Cavin L., Longbottom, A. & Richter. M. (eds), Fishes and the Break-up of Pangaea. Geological Society, London, Special Publications, 295.

Cockerell, T. D. A. 1925. A Fossil Fish of the Family Callichthyidae. *Science*, 62(1609): 397–398.

del Papa, C., Babot, J., Dahlquist, J., García López, D., Deraco, V., Herrera, C., Bertelli, S., Rougier, G.W., Giannini N.P. 2022. Toward a chronostratigraphy of the Paleocene-Eocene sedimentary record in northwestern Argentina. *Journal of South American Earth Sciences*, 113, 103677.

Gayet, M. 1991. "Holostean" and teleostean fishes of Bolivia. Revista Técnica YPFB 12: 453–494.

Gayet, M. and F.J. Meunier. 1998. Maastrichtian to early late Paleocene freshwater Osteichthyes of Bolivia: additions and comments. pp. 85–11. *In*: L.R. Malabarba, R. Reis, R. Vari, Z. Lucena and C. Lucena [eds.]. Phylogeny and Classification of Neotropical Fishes. Edipucrs, Porto Alegre, Brazil.

Gayet, M., L.G. Marshall, T. Sempere, F.J. Meunier, H. Cappetta and J.-C. Rage. 2001. Middle Maastrichtian vertebrates (fishes, amphibians, dinosaurs and other reptiles, mammals) from Pajcha Pata (Bolivia). Biostratigraphic, palaeoecologic and palaeobiogeographic implications. Palaeogeo. Palaeoclim. Palaeoeco. 169: 39–68.

Gil-Delgado, A., Delclòs, X., Sellés, A., Galobart, A., Oms, O., 2023. The Early Cretaceous coastal lake Konservat-Lagerstätte of La Pedrera de Meià (Southern Pyrenees). *Geologica Acta*, 21 (3): 1–18.

Harrison, T., Msuya, C.P., Murray, A.M., Fine Jacobs, B., Báez, A.M., Mundil, R., and Ludwig, K.R. 2001. Paleontological investigations at the Eocene locality of Mahenge in north-central Tanzania, East Africa. pp. 39–74. *In* G.F. Gunnel (ed), Eocene biodiversity: unusual occurrences and rarely sampled habitats, Topics in Geobiology. Kluwar Academic–Plenum Publishers, New York, Vol. 18.

Liu, J., Chang, M., Zhang, J., Chen, G., & Shi, C. (2024). Taxonomic revision and type specimens' location of jianghanichthyids (Ostariophysii: Cypriniformes) from Buxin Formation, Sanshui Basin, China. *Journal of Vertebrate Paleontology*.

Longbottom, A. 2010. A new species of the catfish *Nigerium* from the Palaeogene of the Tilemsi Valley, Republic of Mali. Palaeontology 53: 571–594.

Lundberg, J.G., Sullivan J.P., Rodiles-Hernández R. and Hendrickson D.A. 2007. Discovery of African roots for the mesoamerican Chiapas catfish, *Lacantunia enigmatica*, requires an ancient intercontinental passage. *Proceedings of the academy of natural sciences of Philadelphia*, 156: 39–53.

Malabarba M.C. 2004. On the paleoichthyofauna from the Aiuruoca Tertiary Basin, Minas Gerais State, Brazil. *Ameghiniana* 41(4): 515–519.

Malabarba, M.C.S.L. 1998. Phylogeny of fossil Characiformes and paleobiogeography of the Tremembé Formation, São Paulo, Brazil. pp. 69–84. *In* L.R. Malabarba, R.E. Reis, R.P. Vari, Z.M.S. Lucena, C.A.S. Lucena (eds), Phylogeny and Classification of Neotropical Fishes, Edipucrs, Porto Alegre.

Mirande, J.M. 2018. Morphology, molecules and the phylogeny of Characidae (Teleostei, Characiformes). *Cladistics*, 35 (3): 282–300.

Mo, T. 1991. Anatomy, relationships and systematics of the Bagridae (Teleostei: Siluroidei) with a hypothesis of siluroid phylogeny. Theses Zoologicae 17, Koeltz Scientific Books, Koenigstein, 216 pp.

Muizon C. De & Ladevèze S. 2020. Cranial anatomy of *Andinodelphys cochabambensis*, a stem metatherian from the early Palaeocene of Bolivia. *Geodiversitas*, 42 (30).

Murray, A.M. and L.A. Budney. 2003. A new species of catfish (Claroteidae, *Chrysichthys*) from an Eocene crater lake in East Africa. Canadian J. Earth Sc. 40: 983–993.

Near, T.J., Dornburg, A., Friedman, M. 2014. Phylogenetic relationships and timing of diversification in gonorynchiform fishes inferred using nuclear gene DNA sequences (Teleostei: Ostariophysi). *Molecular Phylogenetics and Evolution*, 80 (2014): 297–307

Otero, O., Cavin L., Cespedes R. In press. South American fossil catfishes: Overview of Siluriformes from the Late Cretaceous - Paleocene of Bolivia. *In* Catfishes, A highly diverse group, Vol. 2, Arratia G & Reis R.E. (eds).

Otero, O. In press. African fossil catfishes. *In* Catfishes, A highly diverse group, Vol. 2, Arratia G & Reis R.E. (eds).

Otero, O., A. Pinton, H. Cappetta, S. Adnet, M. Salem, X. Valentin, J.J. Jeager. 2015**.** A fish assemblage from the Middle Eocene from Libya (Dur At-Talah) and the earliest record of modern African fish genera. PLoS ONE 10: e0144358.

Peyer, B. 1928. Die Welse des ägyptischen Alttertiärs nebst einer kritischen Ubersicht über alle fossilen Welse. Abh. Bayer. Akad., Math.-Natur. Abt. 32: 6–61.

Rabosky D.L., Chang J., Title P.O., Cowman P.F., Sallan L., Friedman M., Kaschner K., Garilao C., Near T.J., Coll M., Alfaro M.E. (2018). An inverse latitudinal gradient in speciation rate for marine fishes. Nature, 559(7714), 392–395.

Sullivan, J. P., J. G. Lundberg and M. Hardman. 2006. A phylogenetic analysis of the major groups of catfishes (Teleostei: Siluriformes) using *rag1* and *rag2* nuclear gene sequences. *Molecular Phylogenetics and Evolution* 41: 636–662.
