# Supplementary figures and images for: Early Biogeography of Otophysi Points to the Neotropics as the Cradle of Characiphysan Fishes
Source: Ecol Evol. 2025 Nov 15;15(11):e72431. doi: 10.1002/ece3.72431 (PMC12619110; doi:10.1002/ece3.72431)

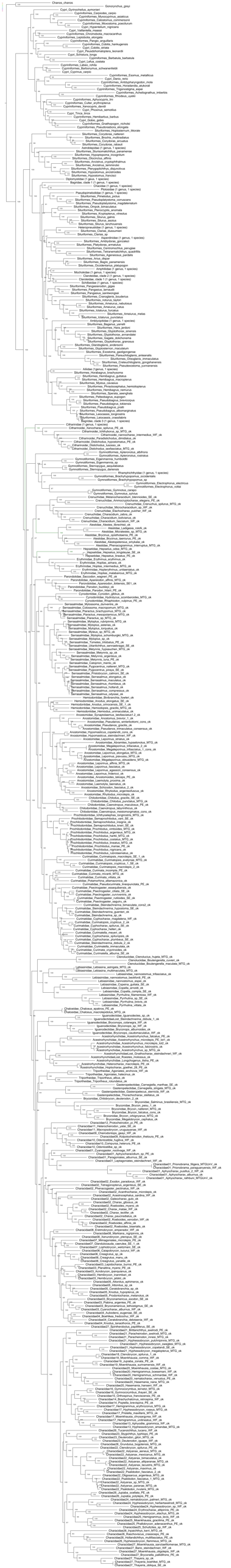

Supplement: Supplementary file 2 — Figure S1: Phylogenetic reconstruction based on mitochondrial genomes with bootstrap scores derived from 5000 replicates. [file ECE3-15-e72431-s009.pdf]

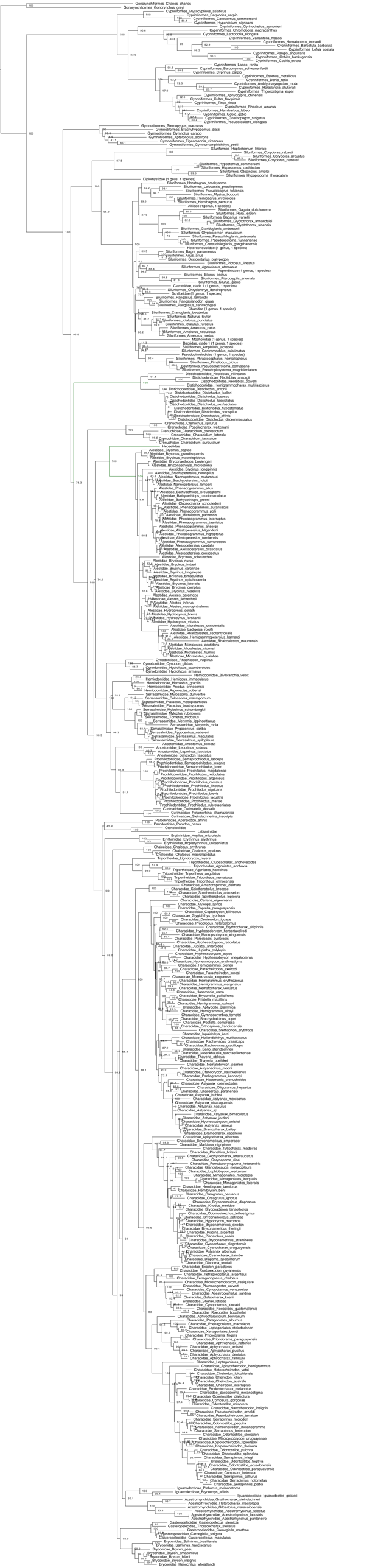

Supplement: Supplementary file 3 — Figure S2: Phylogenetic reconstruction based on four nuclear genes with bootstrap scores derived from 5000 replicates. [file ECE3-15-e72431-s002.pdf]

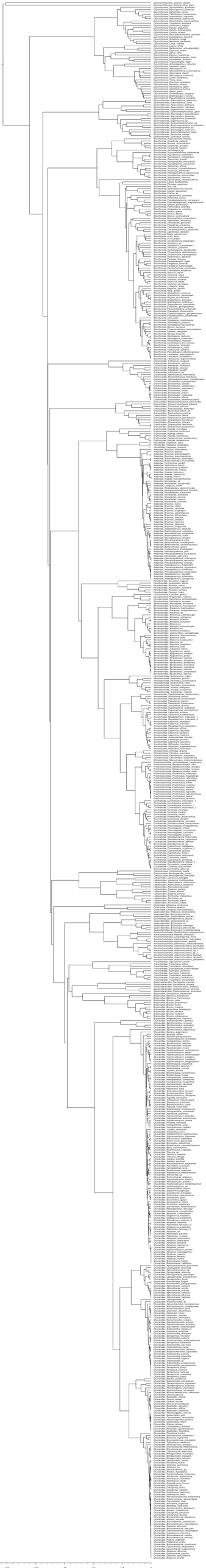

Supplement: Supplementary file 5 — Figure S4: Time tree reconstructed with the discrete model and λ = 10. [file ECE3-15-e72431-s007.pdf]
