# Supplementary material for: Early Biogeography of Otophysi Points to the Neotropics as the Cradle of Characiphysan Fishes
Source: Ecol Evol. 2025 Nov 15;15(11):e72431. doi: 10.1002/ece3.72431 (PMC12619110; doi:10.1002/ece3.72431)

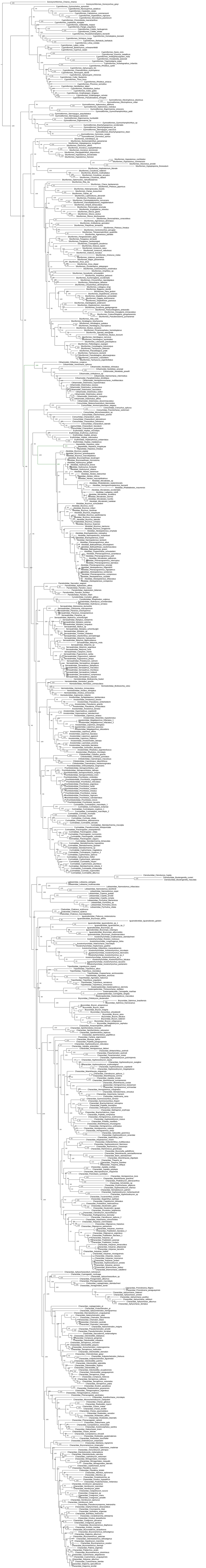

Supplement: Supplementary file 4 — Figure S3: Phylogenetic reconstruction based on the concatenated mitochondrial genomes and four nuclear genes with bootstrap scores derived from 5000 replicates. [file ECE3-15-e72431-s004.pdf]
